# Lower abdominal gossypiboma mimics ovarian teratoma: a case report and review of the literature

**DOI:** 10.1186/s12957-016-1082-3

**Published:** 2017-01-06

**Authors:** Hao Zhang, Yanyong Jiang, Qingqing Wang, Jun Liu

**Affiliations:** 1 Department of General Surgery, The First Hospital of Jiaxing, Jiaxing, Zhejiang 314001 China; 2 Department of General Surgery, Puding County People’s Hospital, Anshun, Guizhou 562100 China

**Keywords:** Gossypiboma, Teratoma, Diagnosis

## Abstract

**Background:**

Gossypiboma is a serious and potentially dangerous medico-legal problem.

**Case presentation:**

We present a case of lower abdominal gossypiboma that presented as an abdominal cystic mass mimicking ovarian teratoma. The mass and the adhesive intestine loop were en blocly resected. The cut section confirmed gossypiboma diagnosis.

**Conclusions:**

The present experience and related literature results indicate that gossypiboma should always be kept in mind for the differential diagnosis of cystic soft-tissue mass detected in patients with a prior operation history despite its rarity and diagnosis difficulty. Once detected or suspected, appropriate surgical intervention should be performed promptly. Most importantly, preventing is much more crucial than curing in order to avoid this highly undesired potential complication.

## Background

Gossypiboma is referred to as a surgical gauze or towel retained inadvertently in the human body during surgery and the resulting reactions. Although it is a rare and preventable occurrence, this challenging medical situation which may induce considerable morbidity and at times even mortality, is a serious and potentially dangerous medico-legal problem [1, 2]. Herein, we report a case of lower abdominal gossypiboma after a cesarean section presenting as an abdominal cystic lump mimicking ovarian teratoma and review of related literature.

## Case presentation

**Fig. 1:**
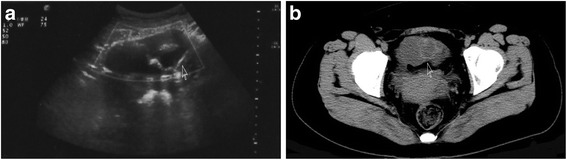
**a** Ultrasound graph revealed echogenic foci and linear reticular echogenic structures. **b** CT scan revealed a lower abdominal cystic mass with hyperdense linear components

A 32-year-old woman was admitted to our hospital Obstetrics and Gynecology Department with intermittent left lower abdominal pain about half month. She had undergone cesarean section through abdominal incision at a suburban maternity hospital 8 years earlier. The results were uneventful. In the follow-up, the patient suffered sometimes from mild intermittent left lower abdominal pain, but eased 1–2 days without any treatment, the problem was explained with postoperative adhesion, and not performed further advanced examinations. She denied any history of fever, vomiting, constipation, or body weight loss at that stage. On physical examination, a lower midline laparotomy scar was noted; a smooth, round, mobile, nontender mass was palpable in the left hypochondrium. On bimanual pelvic examination, a 5 cm × 8 cm mass was felt in the left adnexa region. Blood tests including tumor marker carcino-embryonic antigen (CEA), carbohydrate antigen 199 (CA199), and carbohydrate antigen 125 (CA125) were within normal values. Ultrasound detected a well-encapsulated cystic mass in the left lower quadrant. The lesion showed echogenic foci and multiple linear reticular echogenic structures within it, without blood flow (Fig. 1a). Noncontrast computerized tomography (CT) scan revealed a well-defined, heterogeneous, bulky lesion consisted of hypodense cystic and hyperdense linear components, abutting the adjacent small bowel and sigmoid colon (Fig. 1b).

**Fig. 2:**
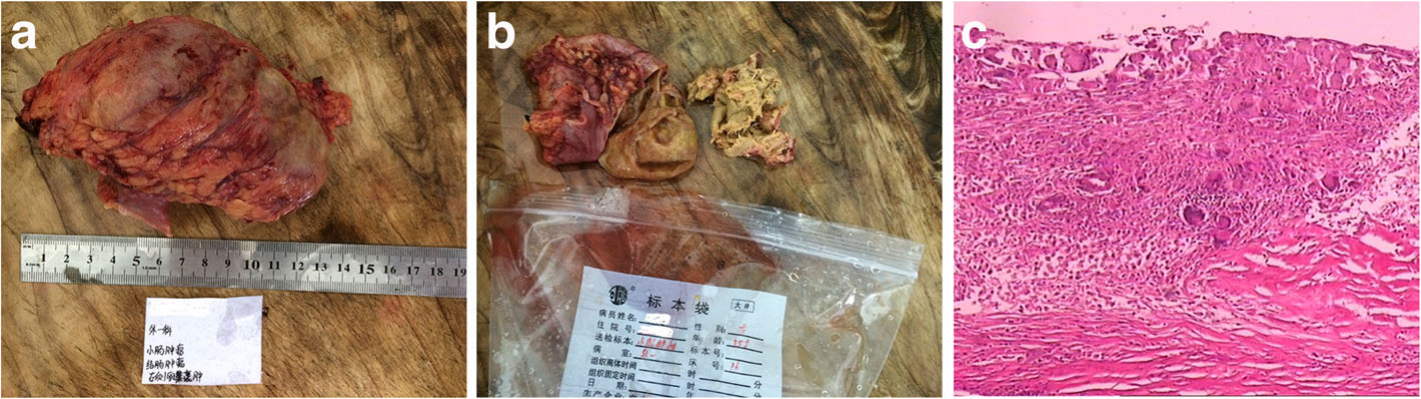
**a** Image of the en bloc-resected specimen with part of the affected intestine. **b** Cut section of the mass showed disintegrated retained surgical sponge. **c** The histological findings demonstrated fibrous encapsulation and foreign body giant cells reaction (×40)

The patient was optimized and taken up for elective laparoscopic exploratory operation. Upon operation, a well-circumscribed firm mass was found, which was about 6 cm × 5 cm, densely adhered to the adjacent greater omentum, sigmoid colon, and intestine, and could not be removed by laparoscopic operation. The uterine and bilateral ovaries were normal except a 3.7 cm × 3.5 cm right ovary cyst. After enucleating the right ovary cyst and consulting the general department surgeon, the operation stopped. The patient was transferred to general department, and laparotomy via a prior surgical incision line under general anesthesia after bowel preparation was performed. At operation, adhesive omentum and sigmoid colon were separated, and the small bowel was too dense to separate, so the densely adhesive intestine loop and the mass were en blocly resected (Fig. 2a). The cut section demonstrated a surgical sponge without a radiopaque marker and yellowish, amorphous liquid material embedded within the fibrotic tissue (Fig. 2b). The histological findings demonstrated fibrous encapsulation containing a large number of foreign-body giant cells reaction (Fig. 2c). The patient was discharged on the 8th postoperative day with uneventful postoperative course and remained symptom-free at 6 months follow-up.

### Discussion

Gossypiboma, otherwise known as textiloma, is a rare incidents caused by retained postoperative foreign bodies. The reported incidence varies between 1 in 1000 and 1500 of all intraperitoneal operations. Probably, the condition is underestimated owing to diagnostic difficulties and medico-legal implications associated with it [3, 4]. Gossypiboma can be observed after all surgical intervention; however, it is more common in the department of general surgery and gynecology surgery at a ratio of 52 and 22% separately [5]. Cholecystectomy has been most commonly associated with the complication, followed by cesarean section and hysterectomy [2]. Women are especially exposed to high risk (63%) since gossypiboma often occurs after gynecological surgery [3, 6].

Gossypiboma clinical manifestation varies and is strongly related to the body reaction as well as the characters of the retained sponge. The patient may present acutely, within months of the original surgery, or may have a delayed presentation years after previous operation. Gossypiboma triggers two types of biological reactions, aseptic fibrotic reaction or exudative inflammatory reaction [7]. The former reaction usually formats an encapsulated mass. Patients usually remain asymptomatic or exhibit nonspecific gastrointestinal symptoms like dull abdominal pain or a palpable painless mass, as well [8–10]. Yet, on the other, exudative inflammatory response induces abscess formation. The disease can be manifested as a serious clinical process presenting with acute abdomen pain and high fever. If not treated timely, may induce bowel or visceral perforation, or even intestinal obstruction, and internal or external fistula formation with adherent organs which may be due to the transmural migration of retained surgical gauzes [11–14].

Gossypiboma usually creates a diagnostic dilemma since clinical symptoms are always not characteristic, and the imaging methods are often uncertain [6, 15, 16]. Despite its rarity and diagnosis difficulty, gossypiboma should always be thought of in the differential diagnosis of indeterminate abdominal pain, infection, or a mass in any postoperative patient. Plain radiography, ultrasonography, and computed tomography (CT) are main imaging methods in establishing the diagnosis.

On plan X-ray, the radiopaque marker attached to the sponge may be easily detected; however, due to the possibility of folding, twisting, or disintegrating over a period of time, the surgical material and marker may be difficult to be identified on a radiograph.

On CT scan, which is the preferred modality, gossypiboma containing gas bubbles and a whorl-like appearance is characteristic. The lesion may appear as a cystic lump with internal spongy appearance mimicking teratoma or dermoid cyst. Occasionally, it may manifest as a hypodense mass, which has a thick peripheral rim and usually misinterpreted as a new-onset tumor or a recurrent tumor [7, 17, 18]. It can be difficult in the diagnosis of a gossypiboma if no radiopaque marker is embedded on the sponge itself.

The typical ultrasonic performance usually presents as a well-defined mass including internal wavy hyperechoic focus, encompassing a hypoechoic rim and having a strong posterior shadow. However, owing to the clinical rarity, this performance is often misinterpreted.

To prevent severe gastrointestinal complications or to overcome the accompanied medico-legal problems, appropriate surgical treatment should be performed as early as possible when gossypiboma is detected or suspected. The most commonly adopted approach is surgical removal through the previous operative site, but treatments like percutaneous, endoscopic, or laparoscopic approaches were also be attempted and reported [19].

Precautions are much more crucial than cure in order to avoid this serious detrimental problem. Strict adherence to surgical material count prior to closing the surgical wound is imperative to avoid the occurrence of this highly undesired potential complication. Surgical materials with radiopaque markers which are useful in reducing the incidence of this condition and making diagnosis in suspected cases should be adopted widely. Although human mistakes cannot be completely eradicated, continuous healthcare staff medical training and vigilant adherence to rules of the operation theaters should reduce the incidence of gossypiboma to a minimum [20]. Gawande et al. reported that emergency surgery, unplanned change in the operation, and BMI are the three significant risk factors prone to inducing the retention of a foreign body [3]. The retention of foreign bodies is generally considered to be avoidable. However, despite the precautions, it still occurs. So, if a high index of suspicion of a foreign body retention residue, further examination is necessary for potential risk although the counting of sponges and instruments is correct at the end of surgery.

## Conclusions

Gossypiboma is a rare and preventable challenging medical situation. If the diagnosis is neglected and not intervene timely, it may cause detrimental impact on patient and the healthcare staff. Once detected or suspected, appropriate surgical intervention should be performed promptly. Most importantly, preventing is much more crucial than curing in order to avoid this highly undesired potential complication.

## Abbreviations

CEA: Carcino-embryonic antigen
CA 199: Carbohydrate antigen 199
CA 125: Carbohydrate antigen 125
CT: Computed tomography
